# Limb Salvage in Methicillin-Resistant Staphylococcus aureus (MRSA)-Complicated Necrotic Loxoscelism Through Lifotronic® Negative Pressure Wound Therapy and Wolfe–McGregor Reconstruction

**DOI:** 10.7759/cureus.101366

**Published:** 2026-01-12

**Authors:** Christopher Kaleb Romero Ríos, Kaleb Mathias Ríos, Francisco Quintana, Roderick Altamirano, Aviezar Blandon

**Affiliations:** 1 School of Medicine, Hospital Militar Escuela "Dr. Alejandro Dávila Bolaños", Managua, NIC; 2 Microsurgery, Clínica Planas, Barcelona, ESP; 3 Surgery, Hospital Antonio Lenin Fonseca, Managua, NIC; 4 Surgery, Hospital Militar Escuela "Dr. Alejandro Dávila Bolaños", Managua, NIC; 5 Orthopaedic Surgery, Hospital Militar Escuela "Dr. Alejandro Dávila Bolaños", Managua, NIC

**Keywords:** loxoscelism, mrsa infection, necrotic spider bite, vac therapy, wolfe–mcgregor skin graft

## Abstract

*Loxosceles reclusa* (brown recluse) envenomation may cause severe necrotic and systemic manifestations, with secondary bacterial infections remaining a major source of morbidity. We report the case of a 69-year-old man with chronic kidney disease, stage 2 hypertension, and prostate carcinoma who developed progressive necrosis of the left hand and forearm following a suspected spider bite. Laboratory findings showed leukocytosis, elevated C-reactive protein, and impaired renal function. Imaging demonstrated subcutaneous fluid collections. Surgical exploration revealed necrosis of the fascia and subcutaneous tissue, while muscular compartments remained viable. Wound cultures grew methicillin-resistant *Staphylococcus aureus* (MRSA). The patient received broad-spectrum antimicrobial therapy with linezolid, ceftazidime, and metronidazole, followed by vacuum-assisted closure therapy using the Lifotronic® Negative Pressure Wound Therapy System and a Wolfe-McGregor skin graft, achieving full recovery of the limb. This case highlights the efficacy of a multimodal approach combining targeted anti-MRSA therapy, negative pressure wound therapy, and reconstructive grafting in achieving limb salvage and preserving functional mobility in high-risk patients with complicated necrotic loxoscelism.

## Introduction

Bites from Loxosceles reclusa remain a medical concern in the Americas, with major clusters described in the southern and central United States and extending into parts of Central America and northern South America. The incidence of clinically significant Loxosceles bites is low, estimated at fewer than 5 000 confirmed cases per year in the United States, although misdiagnosis is common [1-3]. Of those, only 10% advance to dermonecrotic loxoscelism, and systemic manifestations such as hemolysis occur in less than 1% [1]. The venom contains sphingomyelinase D, a necrotizing and proinflammatory enzyme responsible for endothelial injury and tissue ischemia. Severe local reactions predispose to bacterial colonization, which may exacerbate necrosis or lead to bacteremia [2,3]. This report presents a rare case of necrotic loxoscelism complicated by methicillin-resistant Staphylococcus aureus (MRSA) infection, emphasizing the diagnostic complexity and medical-surgical strategies required to avoid disability and systemic sepsis. Given the non-specific nature of the initial lesion, the differential diagnosis is extensive and includes necrotizing fasciitis, Stevens-Johnson syndrome, diabetic ulcers, and cutaneous anthrax. Diagnosis is predominantly clinical and presumptive, relying on the exclusion of these conditions, the patient’s epidemiological context, and the characteristic gravitational progression of the dermonecrotic lesion.

## Case presentation

**Figure 1:**
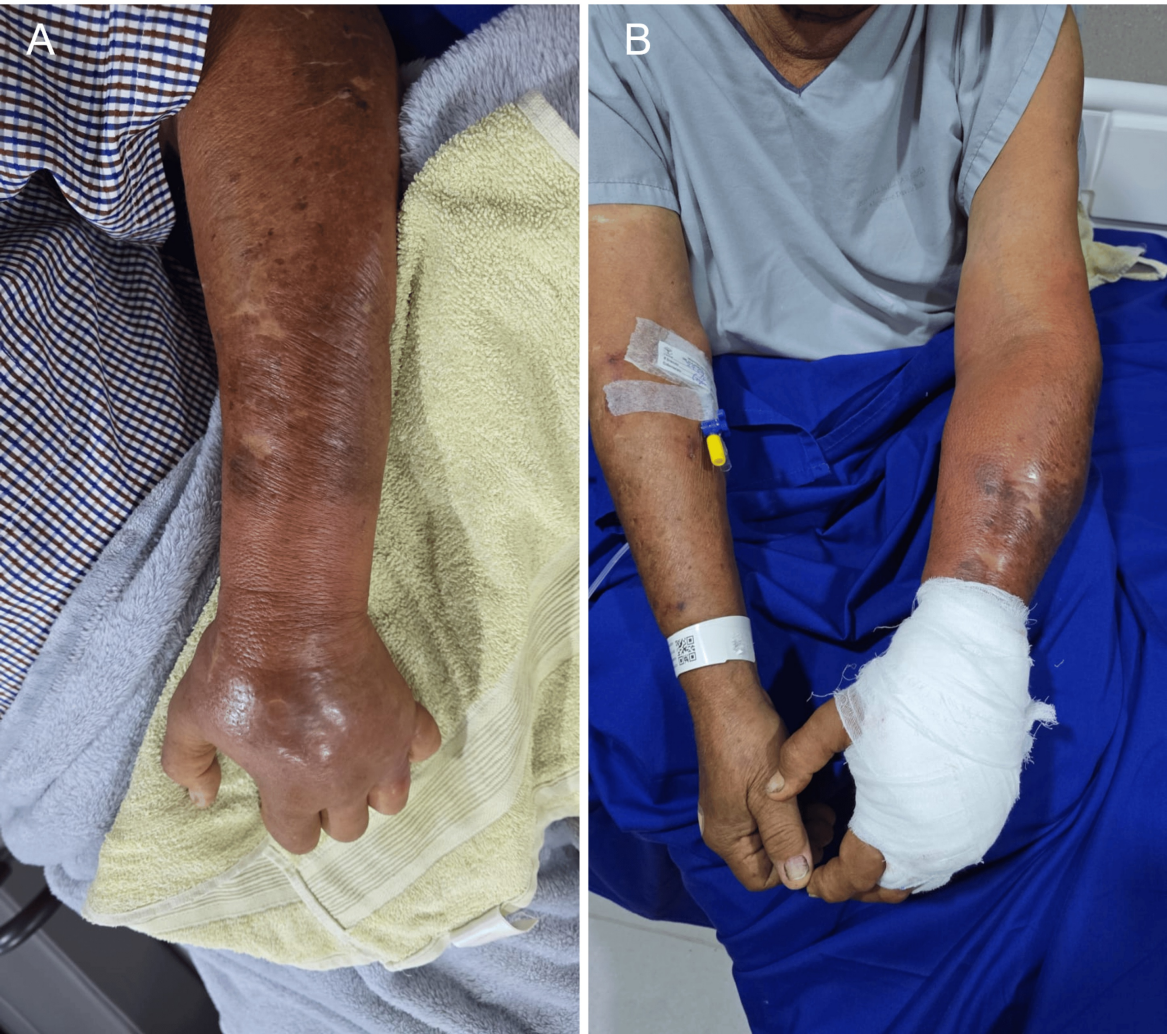
Lesions on the left upper limb. A. Marked edema and erythema. B. Necrotic areas over the forearm.

A 69-year-old man with a history of stage 2 arterial hypertension, chronic kidney disease (KDIGO G2), prostate cancer with biochemical recurrence, and chronic corticosteroid use presented with progressive necrosis of the left hand following a presumptive diagnosis of loxoscelism based on clinical evolution and epidemiological exposure. The patient reported being bitten on the ring finger of the left hand approximately five days prior. Initially, the lesion was neither painful nor symptomatic; however, six hours later, erythema, edema, and a burning pain developed, extending distally and proximally to involve the dorsum of the hand and part of the forearm. On day eight after the bite, he developed fever, malaise, and worsening cutaneous necrosis with violaceous discoloration and purulent exudate (Figure 1).

On admission, vital signs were stable. Physical examination revealed diffuse edema and erythema of the left upper limb. A 5 × 6 cm necrotic ulcer was observed on the dorsal hand, with indurated margins, seropurulent discharge, and firm surrounding edema. Digital flexion was limited by pain on passive movement, but capillary refill remained normal. There were no neurological deficits or findings suggestive of deep necrotizing fasciitis.

**Figure 2:**
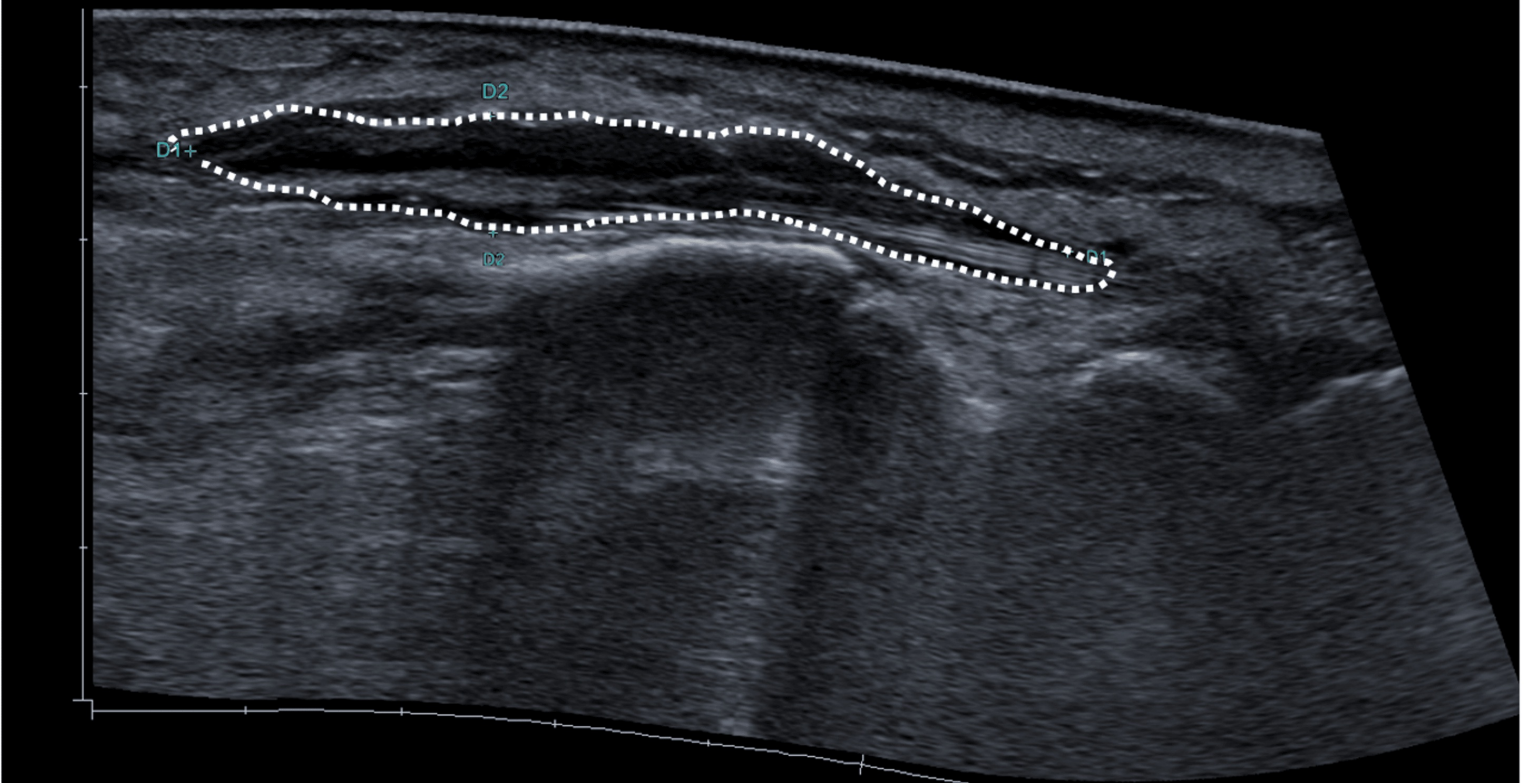
Ultrasound of soft tissues. Significant edema of the subcutaneous tissue in the described area is observed (white lines). Additionally, a hypoechoic, irregularly bordered, avascular lesion is visualized on color Doppler, corresponding to a collection measuring 57 × 7 × 51 mm, with a calculated volume of 11 mL.

Laboratory tests revealed marked leukocytosis (29.75 × 10³/μL; reference range: 4.50 - 11.00 × 10³/μL) with neutrophilia, significantly elevated C-reactive protein (20.2 mg/dL; reference range: < 0.5 mg/dL), and moderate renal impairment (creatinine 1.84 mg/dL; reference range: 0.70 - 1.3 mg/dL). Plain radiography and ultrasound showed soft-tissue swelling with a small subcutaneous abscess measuring approximately 11 mL (Figure 2).

**Figure 3:**
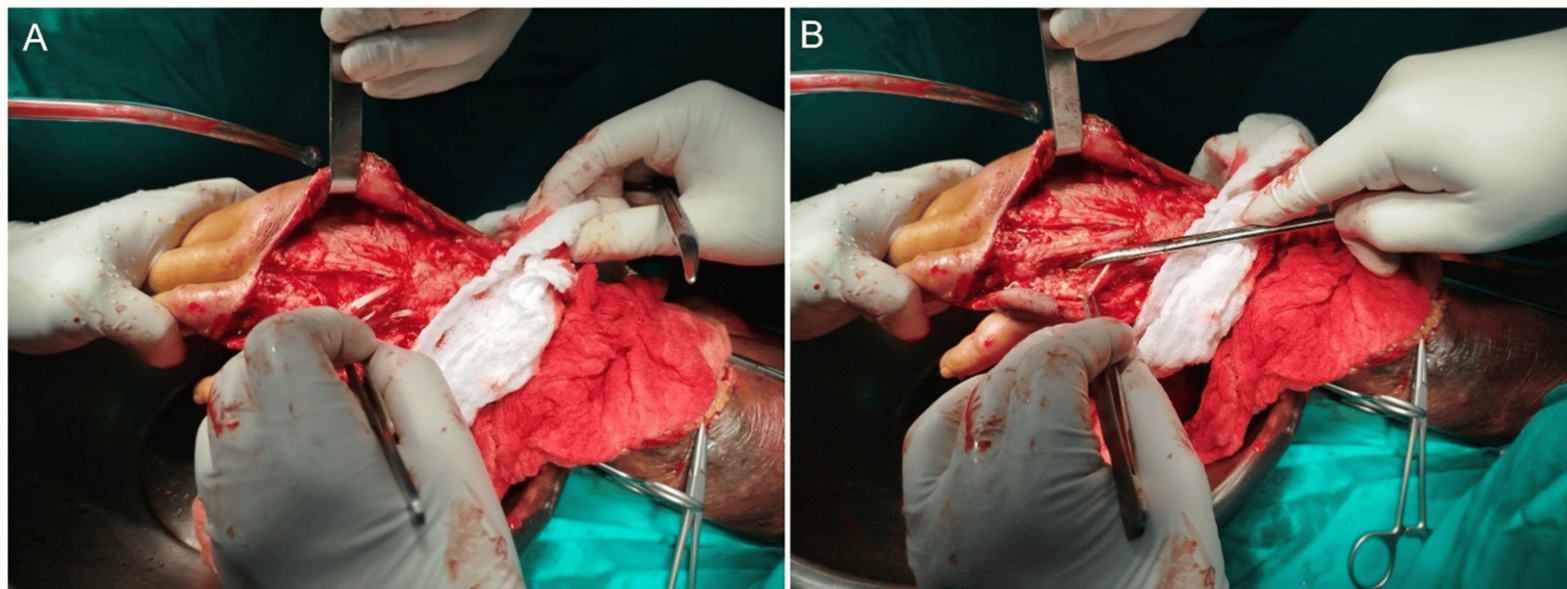
Surgical debridement of the dorsal hand and forearm. A. Intraoperative view of extensor zones III and IV, showing exposed tendon structures following the excision of necrotic subcutaneous tissue. B. Detail of the fourth web space, identified as the initial inoculation site (entry point). Note the infectious spread compromising the adjacent extensor tendon sheaths.

Given the diagnosis of moderate skin and soft-tissue sepsis, empiric intravenous antibiotics were initiated and later tailored to linezolid, ceftazidime, and metronidazole following culture confirmation of MRSA from wound secretions. Surgical management included sequential debridements that revealed subcutaneous and fascial necrosis, with preservation of the forearm and hand musculature (Figure 3).

**Figure 4:**
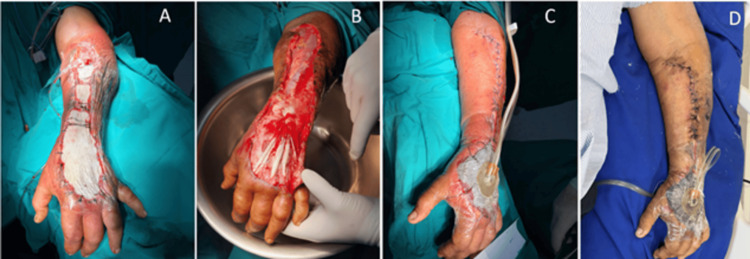
Wound evolution under Lifotronic® VAC therapy. A. First day of VAC application along the left forearm. B. Subsequent days showing healthy granulation tissue and satisfactory progression, particularly over the forearm. C. Skin closure over the forearm with persistent VAC coverage of the dorsal hand. D. Later days showing approximated wound edges and no purulent drainage.\ VAC: Vacuum-assisted closure

A vacuum-assisted closure (VAC) system (Lifotronic® Negative Pressure Wound Therapy) was applied for open wound management and local control of inflammation (Figure 4).

**Figure 5:**
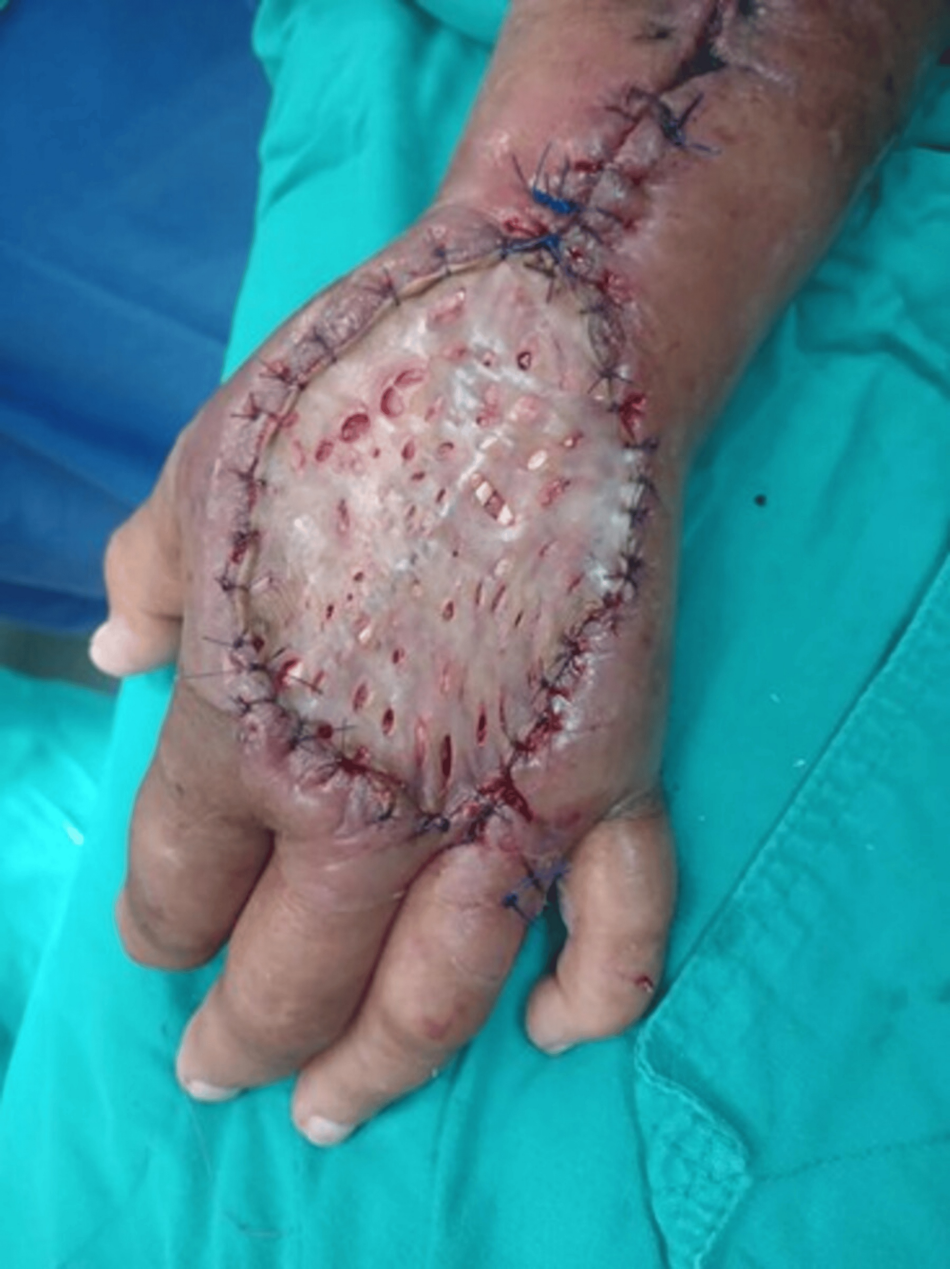
Wolfe–McGregor skin graft over the dorsal hand.

On hospital day 18, a Wolfe-McGregor split-thickness skin graft (5 × 6 cm) was harvested from the right inguinal region to cover the dorsal hand defect (Figure 5).

**Figure 6:**
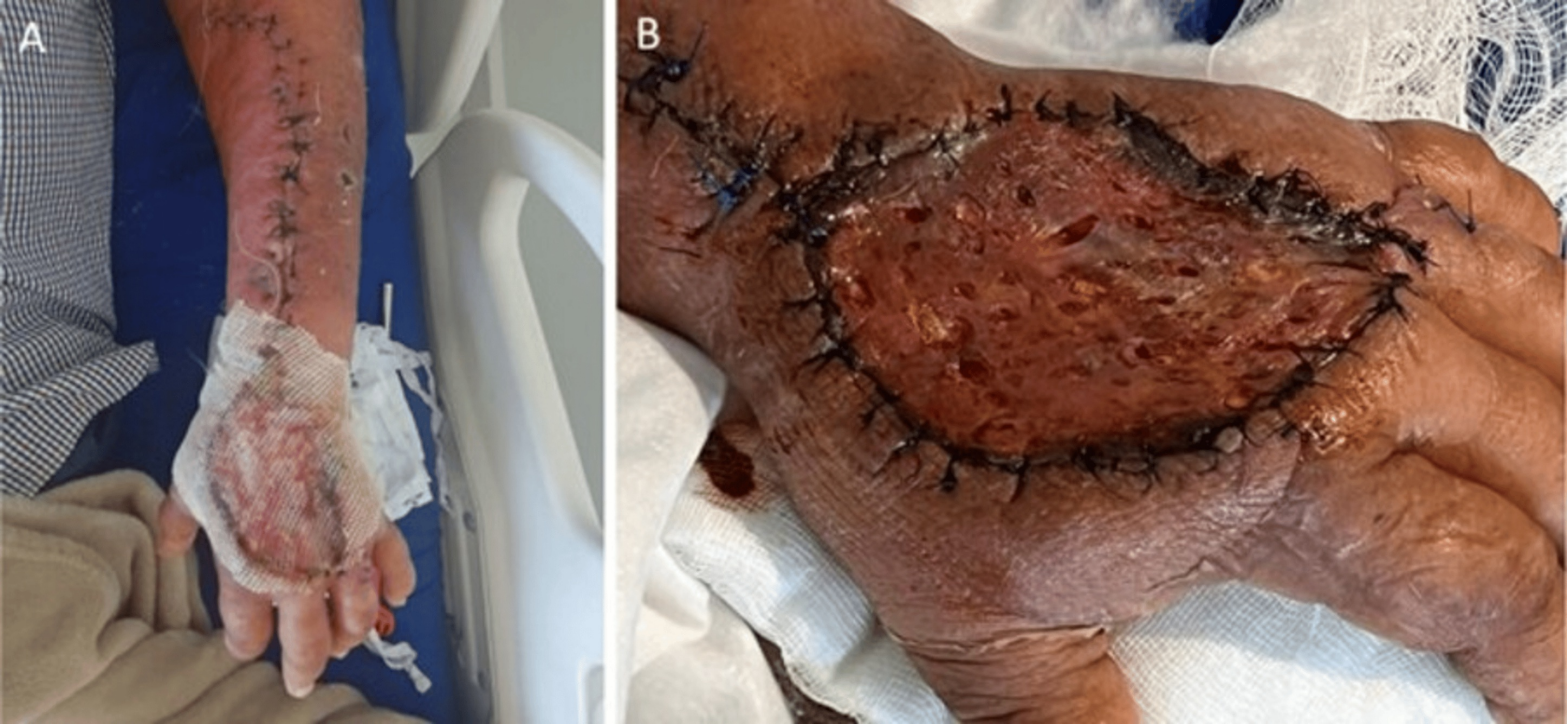
Evolution of the injury. A. Dorsal hand covered with manuka honey-impregnated surgical mesh. B. Favorable 48-hour evolution.

The postoperative course was favorable, with gradual pain reduction, normalization of inflammatory markers, and stable renal function. A subsequent procedure was performed to repair exposed tendons, and a secondary dressing was applied using a honey-impregnated absorbent foam surgical mesh derived from Leptospermum scoparium (medical-grade manuka honey), to accelerate epithelialization and exploit its natural antimicrobial properties. The graft site evolved satisfactorily (Figure 6). Upon discharge, the patient had preserved functional mobility and no signs of active infection. At the three-month follow-up, the functional recovery was satisfactory. The patient demonstrated full extension of the fingers and flexion reaching approximately (90) degrees at the metacarpophalangeal joints, allowing for effective grasp and pincer function without pain.

## Discussion

This report describes an infrequent case of dermonecrotic loxoscelism, the severity of which was drastically magnified by a confirmed superinfection with MRSA. While the literature recognizes that tissue necrosis induced by Loxosceles venom (specifically sphingomyelinase D) creates an ideal avascular bed for bacterial colonization, the coexistence of MRSA represents a primary diagnostic and therapeutic challenge [4]. The venom acts as the initial agent of ischemia, while the secondary infection acts as a potent amplifier of tissue destruction and the risk of systemic sepsis.

The main diagnostic challenge in cases like the present one is differentiating the progression of venom-induced necrosis from the cellulitis or abscesses caused by the bacterial infection [5]. In our patient, the late onset of fever (day 8), marked leukocytosis (29.75 × 10³/μL), and elevated C-reactive protein (20.2 mg/dL) were key indicators that the process had transcended the local inflammation of loxoscelism, evolving into soft-tissue sepsis. This underscores the need for early wound cultures when systemic signs or purulent drainage appear in necrotic lesions attributed to spider bites [6].

The severity of the presentation was also likely influenced by the patient's comorbidities. Chronic corticosteroid use, a known factor in immunosuppression, may have attenuated the initial inflammatory response (facilitating the delay in seeking care) and simultaneously increased susceptibility to an aggressive opportunistic MRSA infection [7]. The pre-existing chronic kidney disease (KDIGO G2), although stable on admission, complicated the selection of antibiotics, requiring careful dose adjustments and the selection of agents with low nephrotoxic potential, such as linezolid, which provided targeted coverage against MRSA [7]. Although wound cultures isolated MRSA, the decision to maintain a broad-spectrum regimen including ceftazidime and metronidazole was driven by the severe necrotic nature of the wound. Necrotic loxoscelism creates an anaerobic environment prone to polymicrobial superinfection that standard superficial swabs may miss. Therefore, covering Gram-negative and anaerobic pathogens was deemed critical to prevent synergistic gangrenous progression in this immunocompromised patient.

Therapeutic success in this case was based on a triple pillar: directed antibiotic control, aggressive surgical debridement, and advanced wound management. Intravenous antibiotic therapy (linezolid, ceftazidime, metronidazole) was crucial but insufficient on its own due to the extensive necrosis. Sequential surgical debridement was essential to remove the devitalized tissue, which acts as an infectious reservoir [8].

Subsequently, negative-pressure wound therapy (NPWT), or VAC, played a crucial role in controlling exudate, reducing edema, and promoting granulation tissue formation over the exposed fascial and tendinous structures [9]. The literature strongly supports NPWT as an effective method for preparing complex wound beds before definitive coverage. In this case, its mechanism of action was twofold: macro-deformation, which approximated the wound edges and reduced defect size, and micro-deformation at the cellular level, which stimulated angiogenesis and granulation. Following NPWT, the application of medical-grade Manuka honey mesh created a low-pH environment that inhibited bacterial proliferation and facilitated autolytic debridement, thereby optimizing the wound bed for graft integration [10]. Finally, the use of a split-thickness skin graft, combined with medical-grade Manuka honey dressings derived from Leptospermum scoparium, was consistent with current wound-closure strategies, leveraging honey’s antimicrobial and epithelialization-enhancing properties to protect the graft and accelerate healing [10].

## Conclusions

Necrotic loxoscelism complicated by MRSA represents a severe diagnostic and therapeutic challenge, particularly when the diagnosis is presumptive and relies on clinical exclusion. This case demonstrates that in immunocompromised patients, a successful outcome requires a broad antimicrobial strategy covering potential polymicrobial and anaerobic co-infections beyond the isolated pathogen combined with aggressive surgical debridement. Furthermore, the integration of advanced adjuvants, specifically negative pressure wound therapy and Manuka honey dressings, proved decisive in preparing the wound bed for grafting. This multidisciplinary approach not only resolved the septic process but ensured limb salvage with preserved functional mobility, preventing permanent disability in a high-risk elderly patient.
